# Urethane Dimethacrylate Influences the Cariogenic Properties of *Streptococcus Mutans*

**DOI:** 10.3390/ma14041015

**Published:** 2021-02-21

**Authors:** Kyungsun Kim, Jeong Nam Kim, Bum-Soon Lim, Sug-Joon Ahn

**Affiliations:** 1 Dental Research Institute and Department of Microbiology and Immunology, School of Dentistry, Seoul National University, Seoul 03080, Korea; kyungsungkim726@gmail.com; 2 Department of Microbiology, College of Natural Sciences, Pusan National University, Busan 46241, Korea; kimjn@pusan.ac.kr; 3 Dental Research Institute and Department of Dental Biomaterial Science, School of Dentistry, Seoul National University, Seoul 03080, Korea; nowick@snu.ac.kr; 4 Dental Research Institute and Department of Orthodontics, School of Dentistry, Seoul National University, Seoul 03080, Korea

**Keywords:** cariogenicity, dental monomer, mutans streptococci, virulence, UDMA

## Abstract

Concerns regarding unbound monomers in dental composites have increased with the increased usage of these materials. This study assessed the biological effects of urethane dimethacrylate (UDMA), a common monomer component of dental composite resins, on the cariogenic properties of *Streptococcus mutans*. Changes in the growth rate, biofilm formation, interaction with saliva, surface hydrophobicity, adhesion, glucan synthesis, sugar transport, glycolytic profiles, and oxidative- and acid-stress tolerances of *S. mutans* were evaluated after growing the cells in the presence and absence of UDMA. The results indicated that UDMA promotes the adhesion of *S. mutans* to the underlying surfaces and extracellular polysaccharide synthesis, leading to enhanced biofilm formation. Furthermore, UDMA reduced the acid tolerance of *S. mutans*, but enhanced its tolerance to oxidative stress, thus favoring the early stage of biofilm development. UDMA did not significantly affect the viability or planktonic growth of cells, but diminished the ability of *S. mutans* to metabolize carbohydrates and thus maintain the level of intracellular polysaccharides, although the tendency for sugar transport increased. Notably, UDMA did not significantly alter the interactions of bacterial cells with saliva. This study suggests that UDMA may potentially contribute to the development of secondary caries around UDMA-containing dental materials by prompting biofilm formation, enhancing oxidative tolerance, and modulating carbon flow.

## 1. Introduction

Dental plaques are generated by a complex and sophisticated community of oral microorganisms. Dietary transitions, especially increased intake of sugars, enhance the proliferation of oral pathogens, resulting in biofilm-associated infectious diseases [1]. Among the oral pathogens, *Streptococcus mutans* is the most important bacteria responsible for dental caries because it promotes biofilm formation, aciduricity, and acidogenicity [2]. In particular, in the initial process of biofilm formation, *S. mutans* attaches to the acquired pellicle on the tooth surface and produces extracellular polysaccharides, which contribute to biofilm development [3].

Dental monomers are categorized into various classes based on their functional groups, which impart specific functions such as adhesion, and have been used as cross-linking agents in composite materials [4]. Among them, bisphenol A glycol methacrylate (bis-GMA) has been used as a matrix for dental restorative materials, a fissure-sealing agent, and a tooth-bonding agent [5]. However, owing to its toxicity and high viscosity, bis-GMA has been replaced with other monomers. Urethane dimethacrylate (UDMA), a dimethacrylate derivative with an ether bond, was developed as an alternative to bis-GMA. Similarly to bis-GMA, UDMA is also highly viscous and is mixed with a low viscosity diluent monomer [6]. Composite formulations are generally a mixture of bis-GMA, UDMA, and diluents in various molecular ratios. Compared to bis-GMA, UDMA has several advantages such as low toxicity, reduced viscosity, and enhanced degree of polymerization, which can increase the degree of conversion, and decrease unreacted monomers in the formulation.

The biocompatibility of dental monomers, specifically their influence on the virulence of oral bacteria, has been of significant concern [7,8,9], because these monomers are known to leach from resin-based materials into the oral environment [10]. Although many studies have extensively evaluated the biological role of bis-GMA in oral microbial ecology, the effects of UDMA have not been fully analyzed. Therefore, this study evaluated the effects of UDMA on the physiology and pathogenesis of *S. mutans*, and the interaction between saliva and *S. mutans*.

## 2. Materials and Methods

### 2.1. Organisms and Growth Conditions

*S. mutans* UA159 was grown in Todd Hewitt (TH) broth (Becton Dickinson, Sparks, MD, USA) at 37 °C in a 5% CO_2_ atmosphere in a Steri-cycle incubator (Thermo Fisher Scientific, Waltham, MA, USA), according to the established procedure. A 2.5 mg/mL solution of UDMA (CAS: 72869-86-4; Sigma-Aldrich, St. Louis, MO, USA) was prepared in dimethyl sulfoxide (DMSO; Sigma-Aldrich, St. Louis, MO, USA). UDMA, which is released from dental materials in the oral cavity, was used at a final concentration of 50 μg/mL, based on previous studies on the leakage of UDMA [11,12]. In a typical experiment, *S. mutans* UA159 that was cultured overnight was diluted 50-fold using fresh TH broth, either with or without UDMA (50 μg/mL), and was grown to the late-exponential phase in a 5% CO_2_ atmosphere at 37 °C in a Steri-cycle incubator for further analyses.

For studying the planktonic growth, a tryptone-vitamin (TV) medium was prepared using 3.5% tryptone (Sigma-Aldrich, St. Louis, MO, USA), 0.2 μg/mL riboflavin (Sigma-Aldrich, St. Louis, MO, USA), 0.2 μg/mL thiamine-HCl (Sigma-Aldrich, St. Louis, MO, USA), 1 μg/mL nicotinamide (Sigma-Aldrich, St. Louis, MO, USA), and 0.04 μg/mL 4-aminobenzoic acid (Sigma-Aldrich, St. Louis, MO, USA), as described previously [13]. The TV medium supplemented with 0.5% of either glucose (Sigma-Aldrich, St. Louis, MO, USA), fructose (Sigma-Aldrich, St. Louis, MO, USA), mannose (Sigma-Aldrich, St. Louis, MO, USA), or sucrose (Sigma-Aldrich, St. Louis, MO, USA) was inoculated with a 1:100 dilution of overnight bacterial cultures and incubated with or without the addition of UDMA (50 μg/mL) at 37 °C. The planktonic growth was monitored photometrically for 48 h at 600 nm using a microplate reader (Infinite F200pro, Tecan, Untersbergstrasse, Grödig, Austria).

### 2.2. Cell Viability Assays

To each well of a 96-well polystyrene microplate (Costar 3595, Corning Inc., Corning, NY, USA) bacterial cells grown in a medium with or without UDMA (50 μg/mL) and a mixture containing phenazine methosulfate (0.03 mg/mL, Sigma-Aldrich, St. Louis, MO, USA) and methylthiazolyldiphenyl-tetrazolium bromide (MTT; 0.5 mg/mL, Sigma-Aldrich, St. Louis, MO, USA) were added in a 4:1 ratio, as previously described [14,15]. After 4 h of incubation at 37 °C, the same volume of a lysis solution containing 50% *N,N*-dimethylformamide (pH 4.7, Sigma-Aldrich, St. Louis, MO, USA) and 20% sodium dodecyl sulfate (Sigma-Aldrich, St. Louis, MO, USA) was added, and the microplate was incubated at room temperature for 16 h. Absorbance values were monitored photometrically at 570 nm using a microplate reader.

### 2.3. Saliva Collection

Unstimulated whole saliva (UWS) was collected from eight healthy volunteers with good oral hygiene into a 50 mL chilled sterile tube using the spitting method. UWS was allowed to accumulate in the mouth floor without eating, drinking, smoking, or brushing teeth 2 h prior to saliva collection. To prepare cell-free saliva, the UWS was centrifuged at 3500× g for 10 min, and the supernatant was filtered through a Millex-GP filter (0.22 μm, Millipore, Darmstadt, Germany). All the volunteers agreed to participate in this study, and the Institutional Review Board approved the protocol (S-D20150031).

### 2.4. Biofilm Formation

Biofilms of *S. mutans* were grown on 96-well polystyrene microplates (Costar 3595, Corning Inc., Corning, NY, USA) with a semi-defined biofilm medium, as described previously [16,17]. After incubation with 100 μL of the UWS at 37 °C for 2 h, each well was rinsed twice with sterilized phosphate-buffered saline (PBS, pH 7.2). Then, a bacterial sample cultured overnight was diluted by 100 times with a pre-warmed biofilm medium containing 20 mM glucose or sucrose, and the cell suspension with or without UDMA (50 μg/mL) was then distributed at 150 μL per well. The plates were then rinsed twice with PBS and air-dried after incubation in a 5% CO_2_ atmosphere at 37 °C for 24 h.

The biofilms were quantitatively evaluated by two different methods: (i) staining, and (ii) determination of colony-forming units (CFUs). In the staining method, biofilms were immersed in 50 μL of a 0.1% crystal violet solution for 15 min and then washed twice. The retained dye was dissolved with 150 μL of acetone:ethanol (1:4) and quantified at 570 nm using a Helios Beta spectrophotometer (Thermo Fisher Scientific, Waltham, MA, USA). In the colony-counting method, biofilms were collected from each well, disrupted briefly by sonication (Q55; Qsonica, Melville, NY, USA), diluted, and then spread on TH agar plates. The plates were incubated at 37 °C for 48 h to allow the development of colonies, and the formed colonies were counted.

### 2.5. Microscopic Analysis 

Biofilms were microscopically examined on eight-well Lab-Tek Permanox chamber slides (Nagle Nunc International, Rochester, NY, USA) using a confocal laser scanning microscope (CLSM; LSM700, Carl Zeiss, Jena, Germany) as previously described [16]. Each well was rinsed twice with Tris-buffered saline (TBS, pH 7.0) and air-dried after incubation with 200 μL of the UWS for 2 h. After the bacterial cultures were prepared in a biofilm medium, as described above, the cell suspensions were distributed at 550 μL per well and incubated with or without the addition of UDMA (50 μg/mL) in a 5% CO_2_ atmosphere at 37 °C for 24 h. Then, the biofilms were visualized by two different methods. In the first method, the extracellular matrices were labeled during the biofilm formation by adding 1 μM Dextran Alexa Fluor^TM^ 647 (Thermo Fisher Scientific, Waltham, MA, USA) to the sucrose-containing medium. After being rinsed twice with TBS, each well was labeled using 10 μM SYTO 13 (Thermo Fisher Scientific, Waltham, MA, USA) for 20 min and rinsed again with TBS. In the second method, a LIVE-or-DIE^TM^ Viability Kit for Bacteria Cells (GeneCopoeia, Rockville, MD, USA) was used to determine the viable cells in the biofilms. The biofilms were stained with TBS containing 0.3% of each staining component, according to the guidelines of the manufacturer. After 2 h of staining in the dark, the plates were rinsed with TBS.

For image acquisition, the biofilms were randomly divided into five areas and each biofilm was scanned using the CLSM. Image capture and two-dimensional projection of z-stacks were performed using ZEN software (version 2.6.76, Carl Zeiss, Jena, Germany), and the image stacks were evaluated using COMSTAT software (version 2.0, MATLAB, Natick, MA, USA).

### 2.6. Cell Surface Hydrophobicity

The cell suspensions grown in a medium with or without UDMA were washed with a PUM buffer (17.4 g of K_2_HPO_4_, 7.26 g of KH_2_PO_4_, 1.8 g of urea, 0.2 g of MgSO_4_ ∙ 7H_2_O, and distilled water to 1000 mL; pH 7.1) [18] and their concentrations were adjusted to an OD_600_ of 0.8 using the same buffer, as described previously [18]. Next, 3.4 mL of the bacterial suspension and 1.7 mL of hexadecane (Sigma-Aldrich, St. Louis, MO, USA) were mixed for 60 s, and the suspension was allowed to settle for 15 min. The optical density of the aqueous phase at 600 nm (OD_600_) was measured using a Helios Beta spectrophotometer (Thermo Fisher Scientific, Waltham, MA, USA). The percentage hydrophobicity was calculated as follows: ((OD_600_ before adsorption−OD_600_ after adsorption)/OD_600_ before adsorption) × 100.

### 2.7. Bacterial Adhesion

For bacterial adhesion assays, cells grown in the presence and absence of UDMA were washed with TBS and then resuspended in TBS to an OD_600_ of 0.5. The cell suspensions were then labeled with 10 μM SYTO 13 for 20 min. After that, a polystyrene microplate (Costar 3516, Corning Inc., Corning, NY, USA) was prepared with saliva, as described above, and 150 μL of the labeled cells was added into each well and incubated for 3 h. The wells were subsequently rinsed with TBS, and the optical density was measured using a microplate reader (excitation: 485 nm; emission: 528 nm).

### 2.8. Glucosyltransferase (Gtf) Activities

A strain of *S. mutans* UA159 lacking the ftf gene that encodes fructosyltransferase was used to prepare Gtf enzymes. FTF-deficient strains were generously provided by Professor Robert A. Burne, Department of Oral Biology, University of Florida College of Dentistry (Gainesville, FL, USA). In order to analyze the Gtf activities of cells grown with UDMA, cell cultures of the FTF-deficient strain grown in a medium with or without UDMA were divided into supernatants and pellets after centrifugation at 3500× g for 10 min as previously described [13,19].

To evaluate the cell-free Gtf activity, 3.1 mM NaN_3_ (Sigma-Aldrich, St. Louis, MO, USA) and 1.0 mM phenylmethylsulfonyl fluoride (PMSF; Sigma-Aldrich, St. Louis, MO, USA) were added after the pH of the supernatant was adjusted to 6.8 with KOH. The supernatant was then concentrated 50-fold, dialyzed against a 20 mM potassium phosphate buffer (pH 6.8) containing PMSF and NaN_3_, and reconcentrated by 50-fold using a VIVASPIN20 centrifugal concentrator (Sartorius Stedim, Goettingen, Germany). To measure the cell-associated activity, the cell pellet was resuspended in a potassium phosphate buffer and fully disrupted using a bead homogenizer. The supernatant was then used to measure the cell-associated Gtf activity after the removal of cell debris. 

The Gtf activity in the synthesis of glucans was determined using radiolabeled sucrose, as previously described [16]. Briefly, Gtf preparations were incubated with 0.6 μCi/mL of [^14^C]sucrose (NEC100X; Perkin Elmer, Waltham, MA, USA) at 37 °C for 4 h. The reaction mixture was then spotted on a fiberglass filter and dried. The amount of radiolabeled glucan was assessed using a TopCount scintillation counter (Perkin Elmer, Waltham, MA, USA). The results were standardized using the total protein content determined using a Bradford assay (Bio-Rad, Hercules, CA, USA) using bovine serum albumin standards. The percentage activity at 4 h was calculated using the activity at time zero as 100%.

### 2.9. Sugar Transport Activity

The activity of the sugar-specific phosphotransferase system (PTS) of permeabilized *S. mutans* grown in a medium with or without UDMA was assessed as described previously [16,20]. In brief, cells harvested with or without UDMA were washed twice with a K-Na phosphate buffer (pH 7.2) containing 5 mM MgCl_2_ and resuspended at one-tenth of their original volume with the same buffer. The cell suspension was then permeabilized with 0.05 mL of an acetone/toluene solution (9:1). To the permeabilized cells, a reaction mixture containing 0.1 mM NADH, 10 mM of the desired carbohydrate (glucose, fructose, or mannose), 10 U of a lactate dehydrogenase (Sigma-Aldrich, St. Louis, MO, USA), and 10 mM NaF (Sigma-Aldrich, St. Louis, MO, USA) in phosphate buffer (50 mM, pH 7.0) was added. Then, 5 mM phosphoenolpyruvate (Sigma-Aldrich, St. Louis, MO, USA) was added and the oxidation of NADH was monitored at 340 nm using a Helios Beta spectrophotometer (Thermo Scientific, Madison, WI, USA). PTS activities were standardized to the total protein concentration, as described above.

### 2.10. Glycolytic pH Drop Assay

Glycolytic acidification was assessed as described previously [16,20]. Briefly, *S. mutans* were grown to the late-exponential phase in a medium with and without UDMA, and centrifuged at 4 °C. The cell pellet was washed with cold distilled water, centrifuged, and resuspended in 5 mL of a solution containing 1 mM MgCl_2_ and 50 mM KCl. The pH of the cell suspension was adjusted to 7.2, and the assay was initiated by adding 55.6 mM glucose. The subsequent pH changes were monitored for 60 min using a SevenMulti^TM^ pH meter (Mettler Toledo, Columbus, OH, USA). 

To evaluate the ability of the cells to reduce the pH in the absence of exogenous carbohydrates, cell suspensions were prepared as described above. The pH changes of the cell suspensions were then monitored for 60 min using a SevenMulti^TM^ pH meter (Mettler Toledo, Columbus, OH, USA) without titrating with external glucose.

### 2.11. Stress Assays

To assess the sensitivity to oxidative stress, cells grown in a medium with or without UDMA were first washed with a 0.1 M glycine buffer (pH 7.0; Sigma-Aldrich, St. Louis, MO, USA) and then resuspended in the same buffer. An aliquot (100 µL) of the cell suspension containing 0.2% hydrogen peroxide (*v*/*v*, Sigma-Aldrich, St. Louis, MO, USA) was obtained at 0, 30, 60, and 90 min, and 50 μg/mL of catalase (Sigma-Aldrich, St. Louis, MO, USA) was immediately added to it. The diluted suspensions were spread on TH agar plates and incubated at 37 °C for 48 h to determine the CFUs.

To assess the sensitivity to acid stress, cells grown in a medium with or without UDMA were first washed with a 0.1 M glycine buffer (pH 7.0) and then resuspended in a 0.1 M glycine buffer at pH 2.8. The cell suspension was then continuously stirred, and aliquots of the cells were retrieved at 0, 30, 60, and 90 min, spread on TH agar plates, and incubated for 48 h at 37 °C to determine the CFUs, as described above.

### 2.12. Statistical Analyses

All assays were performed in triplicate and repeated independently three times. The Mann–Whitney U test was used to analyze the differences in the cell viability, biofilm formation, quantitative COMSTAT data of CLSM images, surface hydrophobicity, bacterial adhesion, and sugar transport activity of the cells grown in the presence and absence of UDMA. Repeated measures analysis of variance was used to compare the time-interval differences in stress assays and Gtf activities between the cells grown in the presence and absence of UDMA. P values less than 0.05 were considered statistically significant.

## 3. Results

### 3.1. Influence of UDMA on the Planktonic Growth and Viability of S. Mutans

**Figure 1:**
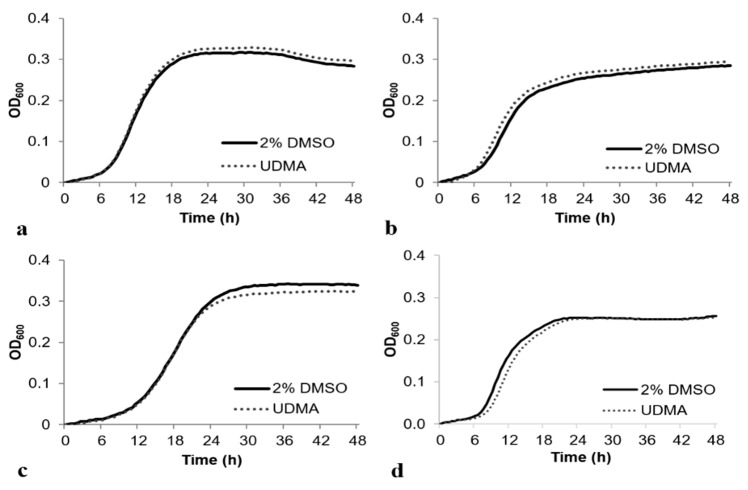
Growth of *Streptococcus mutans* UA159 in the presence and absence of urethane dimethacrylate (UDMA) in a tryptone-vitamin (TV) medium containing 0.5% glucose (**a**), 0.5% fructose (**b**), 0.5% mannose (**c**), or 0.5% sucrose (**d**). DMSO was used as a vehicle for UDMA, and cells grown in the absence of UDMA (2% DMSO) were used as the control.

As the planktonic growth of *S. mutans* in a TH medium with 2% DMSO was not significantly different from that in a TH medium without 2% DMSO (data not shown), 2% DMSO was used for loading UDMA, and 2% DMSO was used as the control for the further analyses. The cell growth was studied in a TH medium containing 0.5% of glucose, fructose, mannose, or sucrose. The cells grown with UDMA (50 μg/mL) demonstrated a sigmoidal growth curve, similar to the cells grown in a medium without UDMA, and the final absorbances of the two samples were comparable (Figure 1). The final absorbance of the cells grown with UDMA in a TV medium containing 0.5% mannose was slightly lower; however, the difference was not significant.

**Figure 2:**
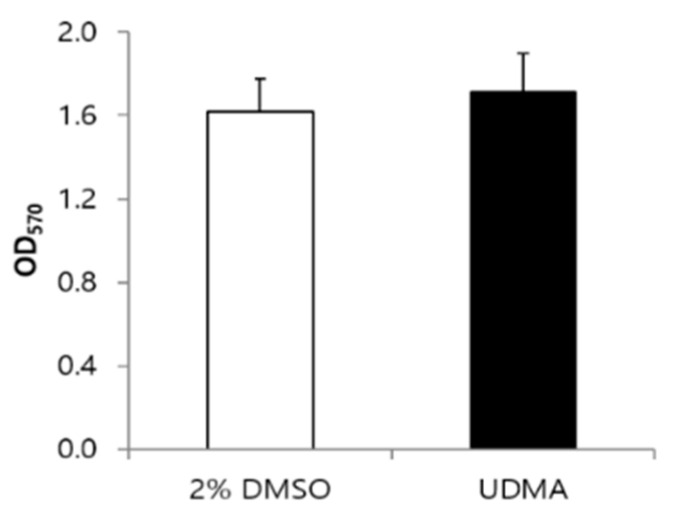
Cell viability of *S. mutans* UA159 grown in the presence and absence of UDMA at 16 h. UDMA was dissolved in DMSO, and cells grown in the absence of UDMA (2% DMSO) were used as the control. The data are expressed as mean ± standard deviation values.

MTT cell viability assays indicated that the viability of cells grown with UDMA was slightly higher, although the difference was not significant compared to the viability of cells grown without UDMA (Figure 2).

### 3.2. Influence of UDMA on Biofilm Formation of S. Mutans

**Figure 3:**
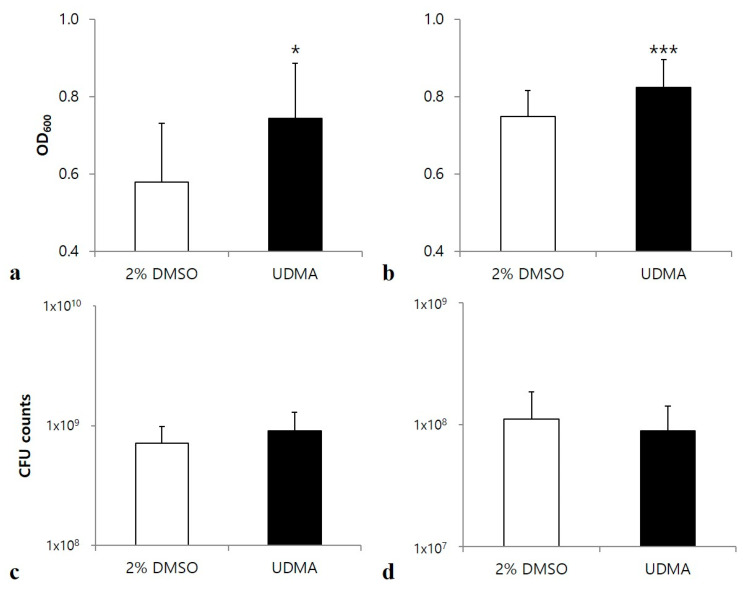
Development of biofilms of *S. mutans* UA159 grown in the presence and absence of UDMA on saliva-coated surfaces for 24 h. Absorbances were determined through the crystal violet assay of biofilms grown in a medium supplemented with glucose (**a**), or sucrose (**b**). Viable cells were counted after the cell growth in a glucose-containing medium (**c**), or sucrose-containing medium (**d**). DMSO was used as a vehicle for UDMA, and the cells grown in the absence of UDMA (2% DMSO) were used as the control. The data are expressed as the mean ± standard deviation values. * *p* < 0.05, *** *p* < 0.001; Mann–Whitney U test.

*S. mutans* biofilms stained with crystal violet indicated increased biofilm development in both glucose and sucrose media in the presence of UDMA (*p* < 0.05 and *p* < 0.001, respectively) (Figure 3a,b).

**Figure 4:**
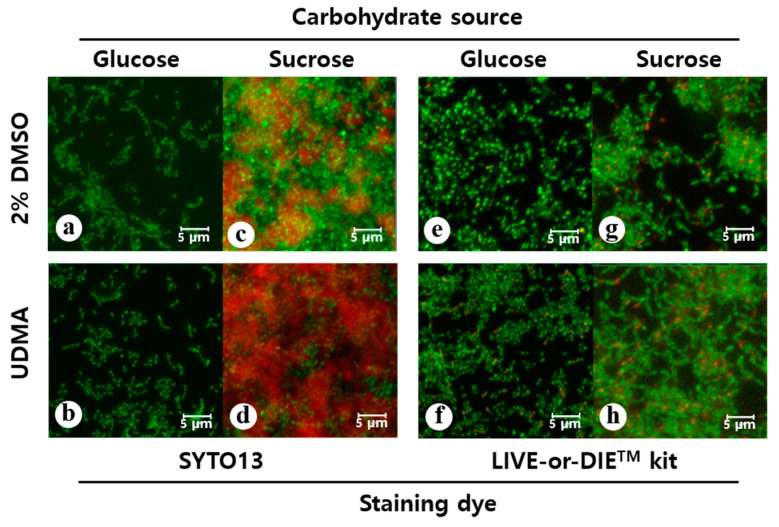
Confocal laser scanning microscopy analyses of the biofilms of *S. mutans* UA159 formed in the presence and absence of UDMA for 24 h (scale bar: 5 µm). To visualize the biofilms, the cells were stained using SYTO13 (green, **a**–**d**) and the polysaccharides were stained using Alexa 647 (red, **c** and **d**). To assess the viable cells, the biofilms were assayed using a LIVE-or-DIE^TM^ viability/cytotoxicity kit (**e**–**h**); in this method, compromised whole cells are stained green and the dead cells are stained red. DMSO was used as a vehicle for UDMA, and the cells grown in the absence of UDMA (2% DMSO) were used as the control. The quantitative fluorescence data are presented in Table 1 and Table 2.

Microscopic images also confirmed that the cell sample grown in the presence of UDMA developed greater amounts of biofilms and contained a remarkably higher number of cells compared to the sample grown in the absence of UDMA (Figure 4a–d). Accordingly, the UDMA-treated cells had higher biovolumes than the control. In particular, cells cultured in sucrose produced more extracellular glucan in the presence of UDMA than in its absence (*p* < 0.05) (Table 1).

Despite the difference in the biofilm formation, no significant difference in the amount of total viable cells was found in the media with and without UDMA, regardless of the carbohydrate source (Figure 3c,d). This was due to the higher number of dead cells detected in the presence of UDMA (Figure 4e–h). As observed in the microscopic images, increased cell death in biofilms was observed in the presence of UDMA, especially in the sucrose-containing medium (*p* < 0.05) (Table 2). These findings indicate that UDMA enhanced the development of the biofilms of *S. mutans*, with increasing cell death.

### 3.3. Influence of UDMA on Cell Surface Hydrophobicity and Bacterial Adhesion

**Figure 5:**
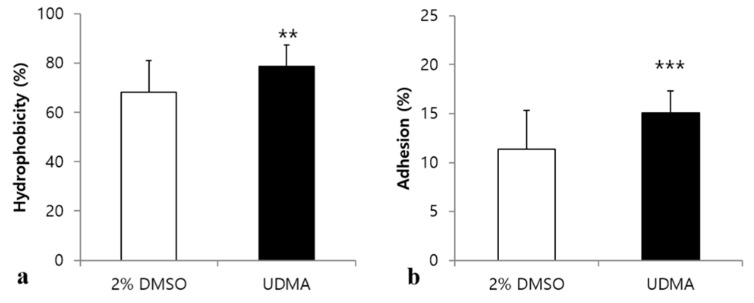
Cell surface hydrophobicity and adhesion of *S. mutans* UA159 treated with UDMA compared to those of the control. The percentages of cells adhered to the hydrocarbon for 15 min settlement (**a**), and saliva-coated surfaces for 3 h (**b**) were determined. DMSO was used as a vehicle for UDMA, and cells grown in the absence of UDMA (2% DMSO) were used as the control. The data are expressed as mean ± standard deviation values. ** *p* < 0.01, *** *p* < 0.001; Mann–Whitney U test.

Bacterial adhesion to biotic and abiotic surfaces is significantly influenced by cell surface hydrophobicity [21]. To determine whether UDMA changes the surface hydrophobicity of *S. mutans*, a hexadecane partitioning assay was conducted (Figure 5a). The results indicated that the cells grown in the presence of UDMA were more hydrophobic than those grown in its absence (*p* < 0.01) (Figure 5a). Subsequently, bacterial adhesion was examined because the changes in surface hydrophobicity are closely related to the ability of *S. mutans* to adhere to the polystyrene surface [21]. The adhesion of *S. mutans* treated with UDMA was significantly enhanced compared to that of the control (*p* < 0.001) (Figure 5b).

### 3.4. Influence of UDMA on the Production of Extracellular Polysaccharides

**Figure 6:**
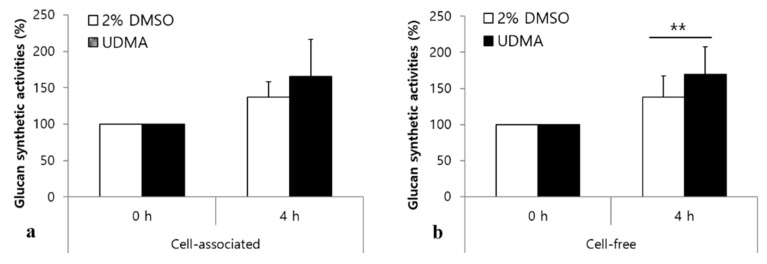
Glucan synthesis activity of *S. mutans* UA159 lacking the *ftf* gene in the presence and absence of UDMA. The relative cell-associated glucan synthesis activity (**a**) was measured in homogenized cell pellets, and the relative cell-free glucan synthesis activity (**b**) was measured in the cellular supernatant. The percentage activities were calculated using the activity at time zero as 100%. DMSO was used as a vehicle for UDMA, and cells grown in the absence of UDMA (2% DMSO) were used as the control. The data are expressed as mean ± standard deviation values. ** *p* < 0.01; repeated measure ANOVA.

Gtfs are enzymes that produce water-insoluble glucans, which promote the tight adhesion of *S. mutans* to tooth surfaces in a sucrose-dependent manner [22]. As shown in Figure 6a, a slight increase in the cell-associated Gtf activity was detected in the presence of UDMA, but the difference in the Gtf activity between the cells grown in the presence of UDMA and those in the absence of UDMA was not significant. However, cells grown in the presence of UDMA exhibited greater cell-free Gtf activity than those in the control (*p* < 0.01) (Figure 6b).

### 3.5. Influence of UDMA on Sugar Transport Activity and Glycolysis

**Figure 7:**
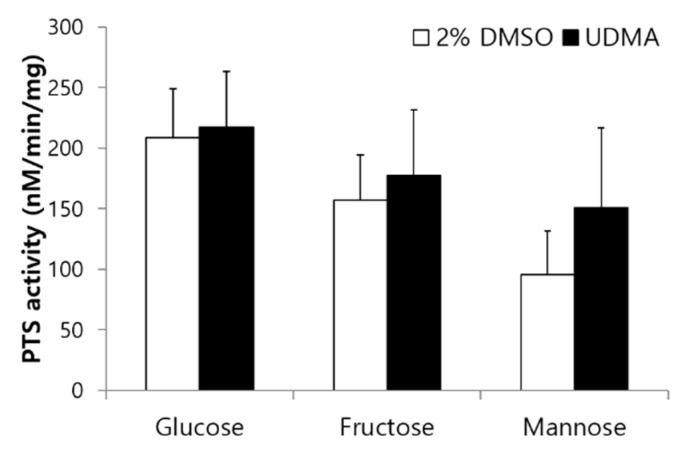
Sugar-specific phosphotransferase activity of *S. mutans* UA159 grown in the presence and absence of UDMA. DMSO was used as a vehicle for UDMA, and cells grown in the absence of UDMA (2% DMSO) were used as the control. The data are expressed as mean ± standard deviation values.

To determine the effects of UDMA on sugar transport modulated by PTS permeases, glucose-, fructose-, and mannose-specific PTS activities were evaluated in the presence and absence of UDMA. UDMA tended to enhance the PTS activity of *S. mutans*, irrespective of the carbohydrate source, but no significant difference was observed between the cells grown in the presence and absence of UDMA (Figure 7). 

**Figure 8:**
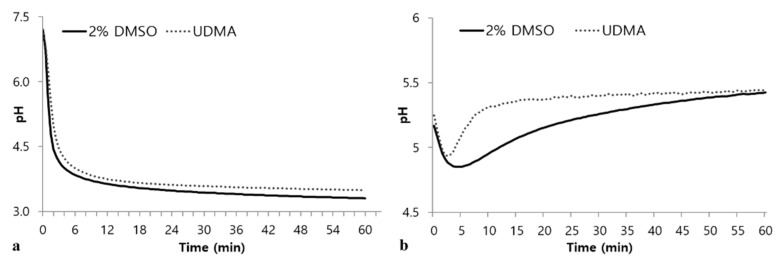
Glycolytic pH profiles of *S. mutans* UA159 grown in a medium with and without UDMA in the presence of excess glucose (**a**), or from endogenous stores (**b**). DMSO was used as a vehicle for UDMA, and cells grown in the absence of UDMA (2% DMSO) were used as the control.

In the glycolytic pH drop assays using glucose as the sole substrate, UDMA-treated cells exhibited retarded glycolysis compared to the control (Figure 8a). In addition, cells grown in the presence of UDMA exhibited a higher final pH than that of the control (Figure 8a). The tendency for a drop pH in the absence of external glucose indicated that cells grown in the presence of UDMA experienced a rapid decrease in pH during the initial stage, followed by a rapid recovery to the final pH (Figure 8b), suggesting that cells grown in the presence of UDMA stored a smaller amount of intracellular polysaccharides than those grown in its absence.

### 3.6. Influence of UDMA on the Acidic and Oxidative Stress Tolerance

**Figure 9:**
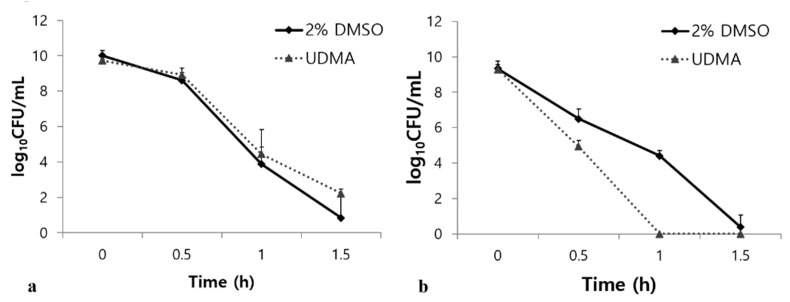
Bacterial responses of *S. mutans* UA159 grown in the presence and absence of UDMA to acidic stress (**a**), and oxidative stress (**b**). DMSO was used as a vehicle for UDMA, and cells grown in the absence of UDMA (2% DMSO) were used as the control.

Tolerance to acid and oxidative stresses are essential characteristics of *S. mutans* for overcoming antagonism by oral commensals and host defenses [23]. Under oxidative stress, UDMA substantially increased the survival rate of the *S. mutans* cells compared to the control (*p* < 0.001, Figure 9a). However, UDMA significantly decreased the stress tolerance of *S. mutans* in an acidic environment (Figure 9b). Acid stress assays revealed that UDMA significantly increased the susceptibility of *S. mutans* to acid stress at pH 2.8 compared to the control cells grown in the absence of UDMA (*p* < 0.001) (Figure 9b). Thus, these results indicated that exposure to UDMA results in a clear reduction in the acid tolerance of *S. mutans*, whereas the tolerance to oxidative stress is enhanced.

### 3.7. Influence of UDMA on the Interaction between S. Mutans and Saliva

**Figure 10:**
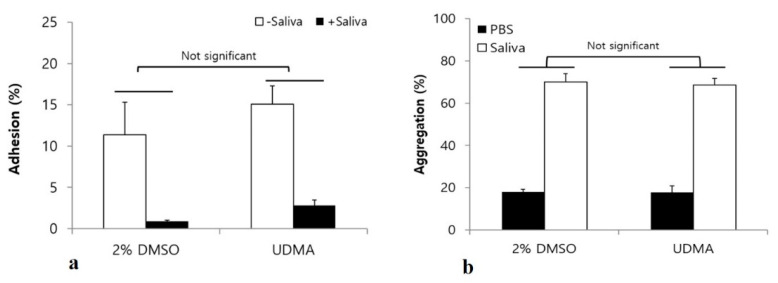
Bacterial adhesion (**a**), and aggregation (**b**) assay to analyze the effects of UDMA on interactions between *S. mutans* UA159 and saliva. Although saliva coating significantly inhibited the adhesion of *S. mutans*, the presence of saliva did not significantly alter the effects of UDMA on bacterial adhesion (two-way analysis of variance). The bacterial aggregation assay indicated no significant difference between the cells grown in the presence and absence of UDMA. DMSO was used as a vehicle for UDMA, and cells grown in the absence of UDMA served as the control (2% DMSO). Data are representative of three independent experiments and are expressed as mean ± standard deviation values.

**Figure 11:**
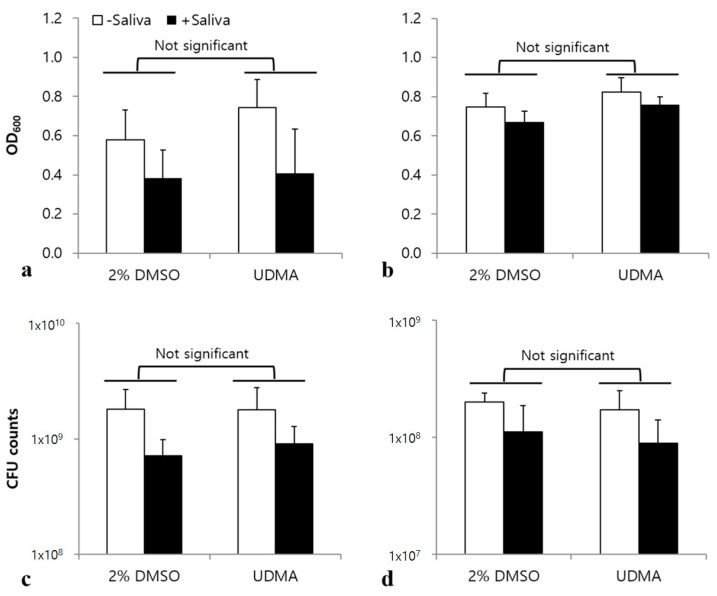
Effects of UDMA on biofilm formation by *S. mutans* UA159 on a surface coated with saliva, and one not coated with saliva in a glucose-containing medium (**a**), and sucrose-containing medium (**b**), as determined by crystal violet staining. Number of viable cells in the glucose-containing medium (**c**), and sucrose-containing medium (**d**). Biofilms were formed in the presence and absence of a saliva coating on the surface. DMSO was used as a vehicle for UDMA, and cells not treated with UDMA served as the control (2% DMSO). Data are representative of three independent experiments, and are expressed as mean ± standard deviation values (Two-way analysis of variance).

Saliva influences the interaction of *S. mutans* with other microorganisms, as well as bacterial adhesion and biofilm development [16]. To verify whether UDMA significantly influences the interaction of *S. mutans* with salivary components, adhesion, aggregation, and biofilm formation were studied in a medium with and without saliva. The results showed no significant differences in bacterial adhesion (Figure 10a), aggregation (Figure 10b), and biofilm development (Figure 11) of *S. mutans* in the presence and absence of saliva, although saliva significantly increased microbial aggregation and inhibited both the adhesion and the biofilm development.

## 4. Discussion

Resin-based dental biomaterials have been extensively used in clinical dentistry because of their excellent performance and superior aesthetic quality [24]. As the use of these materials has increased, concerns regarding the effects of these materials on dental biofilms have also increased because they interact with oral fluids in the oral cavity. This interaction induces dissolution or degradation of the surface layer of the materials, leading to the release of unbound or weakly bound monomers when fluids penetrate the structure of the materials [6,10].

In order to choose target monomers, we investigated the effects of eight leachable monomers (butylated hydroxytoluene, camphor quinone, dimethylaminoethyl methacrylate, ethylene glycol methacrylate, glycidyl methacrylate, bis-GMA, hydroxyethyl methacrylate, methyl methacrylate, triethylene glycol dimethacrylate, and UDMA) on the biofilm formation of *S. mutans* as a pilot study. Among them, only two monomers, bis-GMA and UDMA significantly influenced biofilm formation (data not shown). As the effects of bis-GMA on the cariogenic properties of *S. mutans* have been previously published [6], UDMA was selected as the target material in this study.

Although bis-GMA is widely used as a dental base monomer, UDMA has attracted attention as an alternative material to address concerns about the comparatively higher toxicity and lower degree of conversion of bis-GMA [6,25]. As the strength of intra- and/or inter-molecular hydrogen bonds in UDMA-based resin composites is not sufficiently strong, there is still the possibility of the leakage of free UDMA [26]. In addition, the water solubility of UDMA is higher than that of bis-GMA [27]. Although the effects of bis-GMA on oral microorganisms have been studied extensively [14,25,28], studies on the effects of UDMA are rare. The purpose of this study was therefore to assess the changes in the cariogenic properties of *S. mutans* UA159 exposed to UDMA.

Our data revealed that leachable UDMA significantly increases the biofilm development of *S. mutans*, regardless of the carbohydrate source (Figure 3 and Figure 4, and Table 1). The increased biofilm formation in the presence of UDMA can be explained by the enhanced adhesion of *S. mutans* to hard surfaces (Figure 5b). In addition, it was evident that the increased production of extracellular polysaccharides due to the increased activity of Gtf enzymes in the cell-free fraction led to enhanced biofilm development in the presence of UDMA (Figure 6). This inference is supported by the results of previous studies, which demonstrated that dimethacrylate derivatives, such as UDMA and bis-GMA, increase *gtf* gene expression and Gtf enzyme activity in *S. sobrinus* and *S. mutans* [29,30].

The toxic effects of UDMA on *S. mutans* are evident from the increased number of dead cells in the resulting biofilms (Table 2). This study revealed that the number of viable cells did not increase in the biofilms, despite the increase in the biofilm development in the presence of UDMA (Figure 1 and Table 1). This was verified by the increased ratio of dead cells to whole cells in the biofilms grown in the presence of UDMA (Figure 2e–h and Table 1). These results suggest that *S. mutans* strives to survive against the toxic effects of UDMA. Furthermore, the present study also reveals that *S. mutans* grown in the presence of UDMA tends to show an increased tendency for sugar transport capacity (Figure 7). The working model based on current knowledge is that, in the presence of UDMA, *S. mutans* cells may attempt to route a large portion of carbons to generate additional ATP through fermentation, shifting away from lactate production (Figure 8a). As predicted by this model, although a slightly enhanced activity was detected in the PTS-dependent sugar transport, the extracellular acidification of the cells grown with UDMA decreased in the presence of external glucose (Figure 8a). Furthermore, cells grown in the presence of UDMA had lower amounts of intracellular polysaccharides (IPS) than the cells grown in its absence (Figure 8b). However, these metabolic changes in response to carbohydrate fluctuations were not directly linked to the planktonic growth and cell viability (Figure 1; Figure 2). Thus, these findings support the conclusion that UDMA may enable *S. mutans* to shift its carbon flow toward the ATP generation required for persistence and cariogenicity.

The survival rate of cells grown in the presence of UDMA increased in response to hydrogen peroxide (Figure 9a). When *S. mutans* is exposed to oxidative stress, it is critical to provide a sufficient amount of carbon to balance the ATP generation through the maintenance of the NAD^+^/NADH ratio [13]. Oxidative stress may lead to a significant change in the carbon flow away from the production of lactate. In particular, *S. mutans* shifts its carbon flow toward the generation of additional ATP through the Pta-Ack pathway [31]. This response may partly explain the results of decreased glycolytic acidification and reduced IPS accumulation (Figure 8), as well as the tendency for enhanced PTS activity (Figure 7).

On the other hand, *S. mutans* cells grown in the presence of UDMA were remarkably weak against acid stress (Figure 9b). Many proteins are essential components in the acid tolerance response (ATR) of *S. mutans* during acid stress [32]. In addition, the ATR of *S. mutans* is attributed to other factors, including the fatty acids in the plasma membrane, intracellular alkaline molecules in the cytoplasm, glucan matrix, F_1_F_0_-ATPase activity, and sugar transporters [33,34]. Although the role of F_1_F_0_-ATPase proton pumps is crucial for bacterial survival in response to acid stress, combined defense mechanisms are required to elicit an effective response to acid stress. Our data are supported by previous studies that demonstrated that dimethacrylate-containing monomers, such as bis-GMA, can render *S. mutans* vulnerable to acid stress [14,30]. Considering that exogenous hydrophobic materials affect the fatty acid profile of the plasma membrane in association with the ATR of *S. mutans* [35], it is likely that UDMA can affect the biochemical properties of the membrane by modulating the fatty acid biosynthesis. Although changes in the fatty acid profile were not investigated in this study, the changes in the surface hydrophobicity of the cells and their ability to adhere to surfaces may be correlated with the reduced tolerance to acidic environments. Therefore, our findings provide novel insights into how UDMA induces *S. mutans* to modulate the appropriate balance of carbon flow and membrane properties during oxidative or acid stresses (Figure 9).

Although many studies have examined the mechanical properties and biocompatibility of UDMA [36,37], its effects on the interaction between oral microorganisms and saliva have not been clarified. As salivary glycoproteins, such as mucins and agglutinin, significantly contribute to the adhesion and biofilm formation of *S. mutans* [38], we evaluated the effects of UDMA on the interaction between *S. mutans* and saliva by studying bacterial aggregation, adhesion, and biofilm formation in the absence and presence of saliva. The results revealed that UDMA did not significantly influence the interaction between *S. mutans* and saliva (Figure 10 and Figure 11). Although saliva clearly increased the aggregation of *S. mutans* cells (Figure 10b), and inhibited their adhesion (Figure 10a) and biofilm development (Figure 11), the interactions between *S. mutans* and saliva were not significantly influenced by UDMA. Therefore, the increase in adhesion to hard surfaces and biofilm development may not be due to the effect of UDMA on the interaction of adhesins, which form a part of the cell wall structure, with salivary molecules.

As dental monomers are fundamental components in composite formulations [39], their biocompatibility, specifically their biological effects on oral bacteria, have been investigated extensively [7]. In particular, the effects of bis-GMA on *S. mutans* have been studied in detail, because it is the most commonly used component of resin-based biomaterials [40], and can cause secondary caries as a major side effect [41]. Bis-GMA has been reported to alter the cariogenic properties of *S. mutans* by promoting the IPS accumulation and sugar transport, as well as enhancing biofilm development by increasing glucan synthesis and oxidative tolerance [14]. Similarly to bis-GMA, UDMA also promotes the adhesion of *S. mutans* to saliva-coated surfaces, and extracellular polysaccharide synthesis in *S. mutans*, leading to increased biofilm development. Although UDMA decreased the tolerance of *S. mutans* to acidic conditions, it enhanced the tolerance of *S. mutans* to oxidative stress, thus favoring the early stage of biofilm development and competition with other oral bacteria. In particular, in contrast to bis-GMA, the rapid depletion of IPS in the presence of UDMA indicates that the metabolic flux of *S. mutans* might have changed in favor of its survival, over the storage of IPS for preservation, which is supported by the differences in the planktonic growth and cellular viabilities in the presence of bis-GMA and UDMA. Bis-GMA has been shown to significantly retard the planktonic growth of *S. mutans* and also decrease its viability [14]; in contrast, UDMA did not significantly influence the planktonic growth or cellular viability of *S. mutans* (Figure 1 and Figure 2). UDMA appears to act on biofilm-mediated infections through a mechanism different from that of bis-GMA. 

This is the first report demonstrating that UDMA, a common dental monomer, significantly influenced the cariogenic traits of *S. mutans*, which will provide valuable information on the cause of secondary caries around dental composites in clinical dentistry. However, an in vitro study cannot accurately simulate the clinical environment. Considering that secondary caries might be caused by the leakage of monomers around resin-based dental materials, it is necessary to assess how leachable monomers affect such secondary caries mediated by oral pathogens through further in vivo and in situ studies. 

## 5. Conclusions

The present study demonstrates that leachable UDMA could significantly alter the cariogenic properties of *S. mutans* UA159. The results suggest that UDMA potentially contributes to secondary caries around UDMA-containing dental materials by promoting the adhesion of *S. mutans* to the underlying surfaces, and extracellular polysaccharide synthesis, leading to enhanced biofilm formation. In addition, the presence of UDMA decreased the acid tolerance of *S. mutans*, but enhanced their tolerance to oxidative stress, thus favoring the early stage of biofilm development. Furthermore, UDMA diminished the ability of *S. mutans* to metabolize carbohydrates and thus maintain the level of intracellular polysaccharides, although the tendency of sugar transport increased. These findings clarify the cause of secondary caries around UDMA-containing dental composites in clinical dentistry. As it is impossible to eliminate leachable monomers from dental composites, one of the best methods of preventing secondary caries could be the development of new monomers that show minimum toxicity to host cells, and have antibacterial activity against cariogenic microorganisms.

## Figures and Tables

**Table 1:** Biovolume (µm^3^/µm^2^) of the biofilms of *S. mutans* UA159 formed in the presence and absence of UDMA on saliva-coated surfaces. After 24 h biofilm development, the biofilms were stained with SYTO13 (cells) and Alexa 647 (polysaccharides). DMSO was used as a vehicle for UDMA, and cells grown without UDMA (2% DMSO) were used as the control. The biovolume was calculated based on five randomly selected areas of each biofilm.

Carbohydrate Source	2% DMSO		UDMA	
	Cells	Polysaccharides	Cells	Polysaccharides
Glucose	12.82 ± 1.85	-	13.64 ± 1.21	-
Sucrose	30.06 ± 5.54	5.64 ± 2.76	36.03 ± 6.43	16.11 ± 9.44

**Table 2:** Biovolumes (µm^3^/µm^2^) of the biofilms of *S. mutans* UA159 formed on saliva-coated surfaces in the presence and absence of UDMA, as assayed using a LIVE-or-DIE^TM^ viability/cytotoxicity kit. DMSO was used as a vehicle for UDMA, and cells grown without UDMA (2% DMSO) were used as the control. The biovolume was calculated based on five randomly selected areas of each biofilm after 24 h biofilm development.

Carbohydrate Source	2% DMSO		UDMA	
	Whole Cells	Dead Cells	Whole Cells	Dead Cells
Glucose	3.91 ± 2.47	1.95 ± 1.60	5.75 ± 3.01	2.62 ± 1.90
Sucrose	7.77 ± 4.80	3.09 ± 1.94	13.03 ± 4.46	6.29 ± 2.90

## Data Availability

The data presented in this study are available on request from the corresponding author. The data are not publicly available because it contained personal information.
